# Studies Analyzing South American Public Policy Documents on Physical Activity: A Scoping Review

**DOI:** 10.3390/epidemiologia7040089

**Published:** 2026-06-29

**Authors:** Ingrid Kelly Alves dos Santos Pinheiro, Paulo Henrique Guerra, José Ywgne, Gabriela Fernanda De Roia, Andrea Cortinez-O’Ryan, Danilo Rodrigues Pereira da Silva

**Affiliations:** 1 Laboratory of Kineanthropometry, Physical Activity and Health Promotion, Federal University of Alagoas, Arapiraca 57317-291, Brazil; 2 Health Sciences Graduate Program, Federal University of Sergipe, Aracaju 49107-230, Brazil; danilorpsilva@gmail.com; 3 Institute of Biosciences, São Paulo State University (UNESP), Rio Claro 15385-000, Brazil; 4 Laboratorio de Estudios en Actividad Física (LEAF), Universidad de Flores (UFLO), Buenos Aires C1406AJO, Argentina; 5 Department of Physical Education, Sports and Recreation, Universidad de la Frontera, Temuco 4811230, Chile

**Keywords:** sedentary behavior, scoping review, CAPPA Framework

## Abstract

Objective: To map studies that evaluated national policy documents aimed at promoting physical activity and/or reducing sedentary behavior in South American countries. Methods: A scoping review was conducted, including complete studies that addressed national public policy documents for the promotion of physical activity and reduction of sedentary behavior in any South American country. Eligible studies were those obtained from electronic databases, without restrictions on language or year of publication. The search began with the equation: policy AND (“physical activity” OR “sedentary behavior” OR “screen time”) AND “health” AND (evaluat* OR assess*). Results: Thirteen studies were identified, which allowed us to identify that research conducted in South America has not yet included the analysis of public policy documents aimed at promoting the reduction of sedentary behavior in the subcontinent’s population. The majority of these studies were published in the last four years, and most analyzed Brazilian public policy documents. While some documents from intersectoral initiatives were found, the majority refer to national health sector programs focused on children and adolescents. Conclusion: In addition to helping identify the main characteristics of South American studies analyzing public policy documents on physical activity and sedentary behavior, these findings highlight how little this topic is still explored in the South American subcontinent.

## 1. Introduction

Although the benefits of physical activity have been widely highlighted, its promotion remains a significant challenge. It is estimated that a large portion of the population does not meet the minimum recommendations for physical activity−150 min of moderate-intensity physical activity per week [1], resulting not only in an increase in the development of chronic non-communicable diseases (NCDs) but also in an estimated 5.3 million deaths worldwide. In South America, for example, about 70% of adults meet the physical activity recommendations, and among adolescents, the prevalence of physical activity ranged from 7.5% in Brazil to 19.0% in Suriname [2,3].

In an attempt to reverse this scenario on a global scale, the World Health Organization (WHO), over the past 20 years, has issued guiding documents. One of the most recent, the Global Action Plan on Physical Activity (GAPPA) 2018–2030, has as its main goal to increase the levels of physical activity of the global population by 15% by 2030. To achieve this, it suggests to countries, as the main strategy, the development and implementation of public policies aimed at promoting physical activity for the population [4,5,6]. Following these WHO actions, more than 90% of countries worldwide have been making efforts to develop and implement national physical activity policies [6,7]. Thus, these policies play an essential role in developing actions that promote equity in leisure-time physical activity, enabling people to seek and adopt a physically active and healthy lifestyle [6,8].

Current literature indicates that a public policy of physical activity is indicated by the totality of written formal policies, unwritten formal declarations, written norms and guidelines, formal procedures, and informal policies (or lack thereof) that can directly or indirectly affect physical activity at the community or population level [9]. Nevertheless, it is essential to highlight that policy documents differ from each other, according to their nature. Bull, Milton, and Kahlmeier [10], understanding that the description of some terms may vary among countries, and as a way to standardize the understanding about the definition of what they would be considering as policy (a document that contains priorities, defines goals and objectives, and may or may not contain an action plan), as an action plan (which, being part of a policy or an independent document, determines who, what, when, how, and for how long the planned actions are carried out), and as a program (which, regardless of having a relationship with policies, is a set of measures, with various types of activities linked to its implementation).

In the context of South America, one of the least physically active regions, there are no studies in the literature that offer an overview that comprehensively addresses policies on physical activity and/or sedentary behavior [11], in a way that identifies key concepts, definitions, related factors, gaps in the existing literature, and an understanding of how research is conducted in the area. In other words, it is relevant to map and analyze the evidence in the field of studies on public policies for physical activity in South America, making it possible to analyze how research in the area is being developed, producing information that can support and provide evidence for researchers in the area, managers, and policymakers [12].

Therefore, the objective of this work is to map studies that analyzed national policy documents for promoting physical activity and/or reducing sedentary behavior in South American countries.

## 2. Materials and Methods

A scoping review was conducted using the Joanna Briggs Institute methodological framework [13], registered on the Open Science Framework platform (https://osf.io/8xsca accessed on 27 February 2025), and developed and structured according to the Preferred Reporting Items for Systematic Reviews and Meta-Analysis Protocols (PRISMA-ScR) [14].

To develop the research question, the “population-concept-context” (PCC) [15] mnemonic was used (Population: South American countries, Concept: studies on public policies, and Context: public policies for the promotion of physical activity and reduction of sedentary behavior). Thus, it was possible to formulate the following research question: “What and how have public policies for the promotion of physical activity and reduction of sedentary behavior in South America been analyzed?”.

The scoping review was conducted between July and September 2024, with the inclusion criteria being original studies that address national public policies for the promotion of physical activity and reduction of sedentary behavior in any of the South American countries, without restrictions on language or year of publication. Texts available in the form of abstracts, proceedings, and scientific event programs, as well as any type of review, were excluded.

Evidence sources were obtained from electronic databases: PubMed, LILACS, and Scielo. In the first stage, seeking a broader scope in capturing studies, an initial search was conducted in the PubMed database by testing MeSH terms and index terms, analyzing titles, and abstracts of the retrieved articles to identify possible terms that could be added to the search strategy. In the second stage, the search strategy was adapted to the other databases (LILACS and Scielo), considering the particularities of each. The initial search equation was: policy AND (“physical activity” OR “sedentary behavior” OR “screen time”) AND “health” AND (evaluat* OR assess*). The search strategies used in each of the electronic databases are available in Supplementary Materials Additional File S1. To avoid losing relevant information, we performed a supplementary search on Google Scholar (the first 200 records sorted by date), which did not result in any additional references to the descriptive synthesis.

Data extraction was performed by two independent researchers (IKASP and JY), and in case of disagreement, a third reviewer was consulted (PHG). Rayyan version 1.8 (Rayyan Systems Inc., Cambridge, MA, USA) was used to remove duplicates, select articles by title and abstract, and then evaluate the full text of the study. To assist in this process, a spreadsheet was created in Excel, containing the following information: journal title, author(s), year of publication, country, study objective, policy/program title, policy sector, target population of the policy, strategy, instrument, and main results (Supplementary Materials Additional Files S2–S4). The descriptive synthesis of the data collected was developed based on the refinement of the extraction spreadsheet and was based on the Comprehensive Analysis of Policy on Physical Activity (CAPPA) framework [8] to summarize the interpretation of the main results of the detailed studies, taking into account the indicators: actors, content, context, availability and effect.

## 3. Results

**Figure 1:**
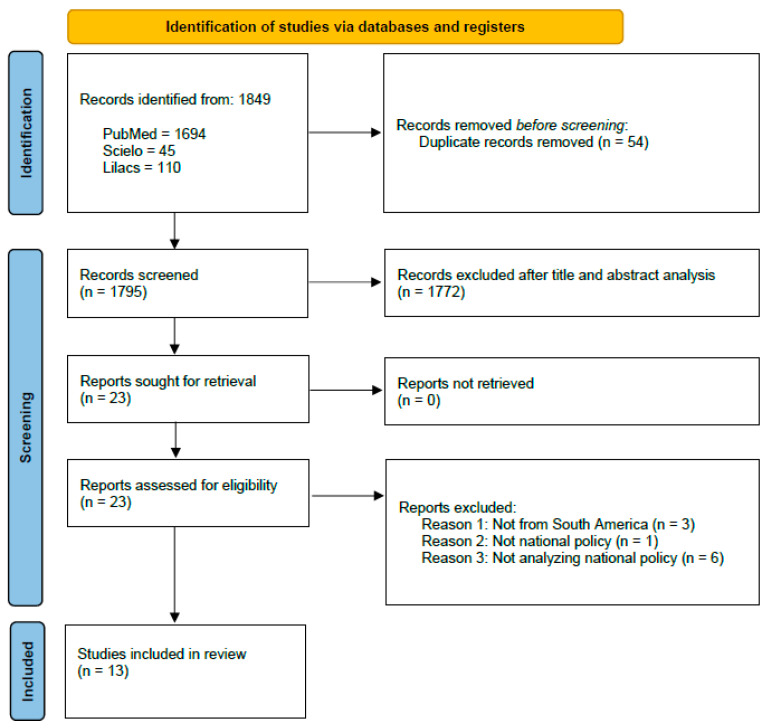
Flowchart of studies throughout the scoping review. Source: Prepared by the authors, 2025.

After applying all the eligibility criteria adopted for this study, and after eliminating duplicates and screening the articles, 13 studies were selected for analysis, as illustrated in the PRISMA flowchart (Figure 1).

As shown in Table 1, most of these were published in the Brazilian Journal of Physical Activity and Health (*n* = 4) [16,17,18,19] and in the Journal of Physical Activity and Health (*n* = 2) [20,21]. It was identified that the majority (*n* = 9) were published in the last four years [7,16,17,18,19,21,22,23,24,25], and that, although some studies analyzed policy documents from Chile, Colombia, and Ecuador, most of the studies (*n* = 9) are about Brazilian documents [16,17,18,19,20,22,23,25,26]. A list with the general characteristics (authors, journal and year of publication, and document title) of each of the studies is presented in Supplementary Materials Additional File S2.

**Figure 2:**
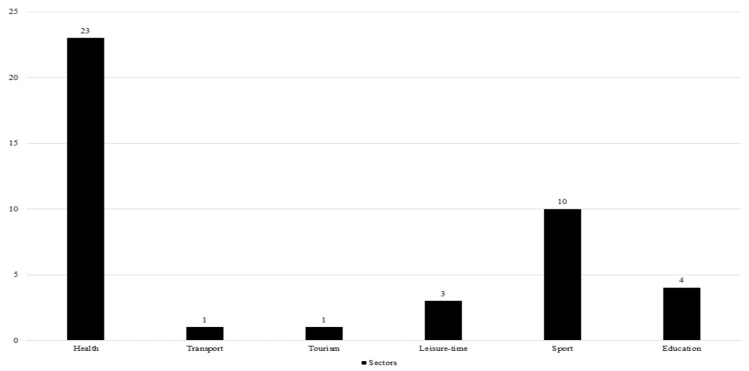
Identification of the sectors of the policy documents that were analyzed by the identified studies (*n* = 25). Source: Prepared by authors, 2025.

None of the selected studies analyzed national policy documents aimed at reducing sedentary behavior; however, among the 13 studies found, a total of 25 national physical activity promotion policy documents were observed (Table 1). With the exception of Knuth et al. [20], who evaluated aspects of a Brazilian national policy, and Grueso et al. [21], who analyzed Colombian decennial plans and national policy and two Ecuadorian decennial plans and one program, all other studies analyzed programs aimed at stimulating physical activity. Furthermore, it is noted that the vast majority of these documents are aimed at children and adolescents (*n* = 17) [19,21,23,25]. Furthermore, although most documents (*n* = 22) encompass the health sector, intersectorality stands out among some [19,21,23,24,25,27], especially among the sports, education, and leisure sectors (Figure 2).

The majority of studies (*n* = 9) adopted strategies to contact people involved, directly or indirectly, with the analyzed policies, through conducting interviews (*n* = 4) [16,17,24,26], applying questionnaires (*n* = 3) [19,20,27], or using specific tools (*n* = 3) [7,21,23], with the obtained responses being analyzed descriptively in most studies (*n* = 8) [16,19,20,21,23,25,26,27] (Table 2). Details on the methodological aspects of each of the studies are available in Supplementary Materials Additional File S3.

**Figure 3:**
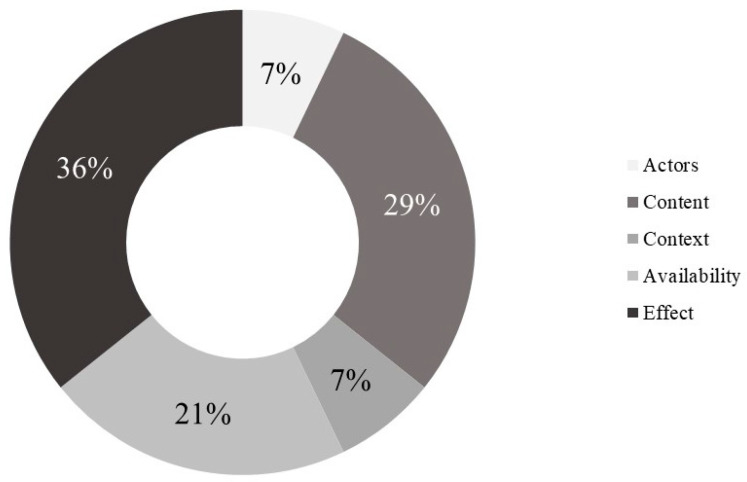
Elements evaluated by studies that analyzed public policy documents on physical activity and/or sedentary behavior in South American countries. Source: Prepared by the authors, 2025.

Based on the information on the objectives and main results of each study, detailed in Supplementary Materials Additional File S4, it can be seen that each of the studies has different objectives and, therefore, ranges from a characterization of policy documents to an analysis of the impact of their respective implementations. That is, it is possible to perceive that they, in large majority, verify the availability [16,19,20], the content [17,23,25,26], or the effects [7,16,18,21,22] of policy documents (Figure 3).

## 4. Discussion

This scoping review aimed to map studies that analyzed national policy documents for the promotion of physical activity and/or reduction of sedentary behavior in South American countries. Thirteen studies were found, which allowed the identification of the following: (1) the majority of publications are recent (within the last four years) and centralized in Brazil, highlighting the Academia da Saúde (Health Academy) and Saúde na Escola (Health in School) programs; (2) research in the region does not yet encompass the analysis of policies aimed at reducing sedentary behavior; and (3) although intersectoral initiatives exist, national health programs targeting children and adolescents predominate.

Although the literature frequently highlights disparities in scientific infrastructure [28], a lack of representativeness in investigations on physical activity policies in low- and middle-income countries [11], and slow progress in the analysis of these policies in the Americas [29], the findings of this research reveal a scenario of scientific resilience in South America. Studies developed in Chile, Colombia, and Ecuador were identified, with special emphasis on Brazil as the regional “epicenter” in the research field and in the publication of guiding documents for national policies on physical activity promotion [22,30]. Generally, as these are the countries in the subcontinent that concentrate the largest volume of physical activity policies, they ratify the existence of a two-way street between scientific production and political practice [31]. Nevertheless, the apparent heterogeneity and comparatively lower representation of other countries in the subcontinent in the literature may also be influenced by structural factors, including differences in research capacity and variability in the indexing of regional journals in major international databases. However, for this scenario to improve, it is necessary to advance the promotion of new research, the availability of national policies for physical activity promotion, and the existence and regularity of national physical activity surveillance systems, as enhancing any of these pillars can lead to improvements across the entire spectrum [31,32].

One important finding from this review was the absence of studies specifically addressing sedentary behavior policies. This may be explained by the fact that sedentary behavior, as a concept distinct from physical inactivity, is relatively recent, particularly in low- and middle-income countries. Smirmaul [33] illustrates the evolution of this field by contextualizing it within human history, highlighting how the discussion of sedentary behavior and its health implications has only recently gained scientific and public health attention. As a relatively novel social phenomenon, the varying definitions applied to sedentary behavior have contributed to conceptual misunderstandings. The lack of clear conceptual standardization compromises the comparison and interpretation of scientific evidence. Thus, it is confirmed that intersectoral collaboration is fundamental for population-wide physical activity promotion strategies to have their importance recognized and to be successful and effective [34,35,36,37]. Furthermore, similar to other studies in this research field, the adoption of statistical procedures that describe and present the characteristics of these documents is common [10,38]. Despite this, research developed in South American countries analyzed national physical activity policy documents from diverse and distinct perspectives, in addition to adopting methodological strategies and instruments that diverge from those adopted by studies developed in other continents [5,28,38,39]. These factors illustrate the challenges that researchers have faced from policy identification to analysis, which, to some extent, may justify the limited progress in this field of study in South America, given that conducting a robust analysis of public policies on physical activity requires the use of instruments, tools, and techniques that aid in their true understanding [39].

The emergence of global guidelines, such as the 2018–2030 GAPPA [4] and the 2020 WHO Guidelines on physical activity and sedentary behavior, aimed at updating and structuring effective and feasible policy actions to increase physical activity [4], may have encouraged the scientific community to examine the current landscape in order to generate evidence to inform future actions, particularly regarding the actual impact of sedentary behavior on health and its role in policy decision-making processes [40]. Although these conceptual and methodological challenges have gradually been addressed, scientific evidence on the prevalence, trends, determinants, and health outcomes associated with sedentary behavior has expanded primarily in high-income countries, especially over the last decade. In contrast, low- and middle-income countries, including those in South America [7,11], have not progressed at the same pace, largely due to insufficient resources and limited funding to support research in this field [41,42].

We also found that the analyzed studies were predominantly linked to the health, sports, and education sectors, aligning with the overview described in the literature [6,7,10,11,43]. In this context, this review shows that programs were the main type of document analyzed. Whether or not they are directly linked to specific policies, these analyses contribute to understanding their impact on population-level physical activity promotion and provide evidence supporting the continuity of implemented interventions and activities [43,44].

This work presents some limitations, such as the absence of searches for gray literature works, such as dissertations and theses, and the implementation of a search strategy limited to the health field, not allowing for comprehensiveness regarding the sectoral distribution of documents. As main strengths of this review, we can cite (1) the search conducted in relevant electronic databases; (2) the use of a rigorous methodological process for the selection of studies; and (3) the most current conduction, and exclusive in South American countries, of studies on the analysis of public policies on physical activity and/or sedentary behavior.

That said, it can be affirmed that this scoping review provides relevant information for researchers interested in the topic, potentially assisting them, particularly, in decision-making regarding methodological strategies to be adopted for conducting future studies. In other words, this review maps how regional research has been conducted by highlighting which document types receive the most attention, uncovering major gaps in current methodological strategies, and demonstrating that public policy analysis remains in an initial stage within the South American subcontinent. From a theoretical perspective, these findings also suggest that the translation of policy instruments into population-level behavioral change is a complex, multilevel process that extends beyond the existence of formal policy documents. It depends on implementation fidelity, intersectoral coordination, and the capacity of health and education systems to operationalize actions in real-world settings, which may help explain why the empirical literature identified in this review tends to focus more on policy adoption and documentation rather than on sustained behavioral outcomes.

## 5. Conclusions

This review points to a research field that is expected to be on the rise, while emphasizing the methodological design necessary to address the highlighted gaps, such as the scope of coverage and representativeness. For future investigations, in addition to recommending the development and utilization of policy analysis tools tailored to the local socioeconomic realities of the subcontinent, we emphasize the strengthening of transnational initiatives to include, support, and foster connections among researchers in South America.

## Figures and Tables

**Table 1:** Characterization of studies that analyzed public policy documents on physical activity and/or sedentary behavior in South American countries.

Journals (*n* = 13)	*n*	%
Caderno de Saúde Pública	1	7.7
Ciência & Saúde Coletiva	1	7.7
Health Promotion International	1	7.7
International Journal of Behavioral Nutrition and Physical Activity	1	7.7
International Journal of Environmental Research and Public Health	1	7.7
Journal of Physical Activity and Health	2	15.3
Revista Brasileira de Atividade Física e Saúde	4	30.7
Revista Médica de Chile	1	7.7
Saúde debate	1	7.7
**Year of Publication (*n* = 13)**	***n***	**%**
2010	1	7.7
2017	1	7.7
2018	1	7.7
2020	4	30.7
2021	1	7.7
2022	3	23
2024	2	15.3
Countries (*n* = 12) *	***n***	**%**
Brazil	9	75
Chile	1	8.3
Colombia	1	8.3
Ecuador	1	8.3
**Type of documents (*n* = 25)**	***n***	**%**
Policies	2	8
Plans	3	12
Programs	20	80
**Population (*n* = 25)**	***n***	**%**
Children and Adolescents	17	68
Women	1	4
General Population	7	28

* The study by Pogrmilovic et al. does not detail which South American countries make up its sample. Therefore, for this indicator, we analyzed 12 studies with available information about the country. Source: Authors of the article, 2025.

**Table 2:** Identification of strategies, instruments, and analyses adopted by studies that analyzed public policy documents on physical activity and/or sedentary behavior in South American countries (*n* = 13).

Strategy	*n*	%
Online search for documents	4	30.8
Contact key informants	3	23.1
Contact policy stakeholders	3	23.1
Contact policy users	3	23.1
**Instrument**	***n***	**%**
Report	3	23.1
Interview	4	30.8
Questionnaire	3	23.1
Tool	3	23.1
**Analysis**	***n***	**%**
Descriptive	8	61.5
Descriptive and Inferential	2	15.3
Descriptive and Qualitative	1	7.7
Inferential	2	15.3

Source: Authors of the article, 2025.

## Data Availability

The original contributions presented in this study are included in the article/Supplementary Materials. Further inquiries can be directed to the corresponding author.

## Supplementary Materials

The following supporting information can be downloaded at: https://www.mdpi.com/article/10.3390/epidemiologia7040089/s1, Additional File S1—Search strategy in electronic databases; Additional File S2—General characteristics of studies that analyzed public policy documents on physical activity and/or sedentary behavior in South American countries; Additional File S3—Methodological aspects of studies that analyzed public policy documents on physical activity and/or sedentary behavior in South American countries; Additional File S4—Objectives and main results of studies that analyzed public policy documents on physical activity and/or sedentary behavior in South American countries.
