# Supplementary material for: Studies Analyzing South American Public Policy Documents on Physical Activity: A Scoping Review
Source: Epidemiologia (Basel). 2026 Jun 29;7(4):89. doi: 10.3390/epidemiologia7040089 (PMC13397982; doi:10.3390/epidemiologia7040089)
Supplement: Supplementary file 1 [file epidemiologia-07-00089-s001.zip › Additional File S1_Scoping review_Ingrid.pdf]

Additional File S1 - Search strategy in electronic databases (search conducted on July 24, 2024)

Pubmed	policy[Title/Abstract] AND ("physical activity"[Title/Abstract] OR "sedentary behavior"[Title/Abstract] OR "screen time"[Title/Abstract]) AND "health"[Title/Abstract] AND (evaluat*[Title/Abstract] OR assess*[Title/Abstract])
Lilacs	policy AND ("physical activity" OR "sedentary behavior" OR "screen time") AND "health" AND (evaluat* OR assess*) AND (db:("LILACS"))
Scielo	policy AND ("physical activity" OR "sedentary behavior" OR "screen time") AND "health" AND (evaluat* OR assess*)
