# Supplementary material for: Studies Analyzing South American Public Policy Documents on Physical Activity: A Scoping Review
Source: Epidemiologia (Basel). 2026 Jun 29;7(4):89. doi: 10.3390/epidemiologia7040089 (PMC13397982; doi:10.3390/epidemiologia7040089)
Supplement: Supplementary file 1 [file epidemiologia-07-00089-s001.zip › Additional File S2_Scoping review_Ingrid.pdf]

Additional File S2 - General characteristics of studies that analyzed public policy documents on physical activity and/or sedentary behavior in South American countries (n=13).

Journal	Author	Year of publication	Country/Contries	Document
Journal of Physical Activity and Health	A. G. Knuth, D. C. Malta, D. K. Cruz et al.	2010	Brazil	National Health Promotion Policy
Caderno de Saúde Pública	R. N. Silva, F. R. B. Guarda, P. C. Hallal, P. J. L. Martelli	2017	Brazil	Health Academy Program
REVISTA MÉDICA DE CHILE	R. Mora, M. Greene, M. Coradoc	2018	Chile	CicloRecreoVia Program
International Journal of Behavioral Nutrition and Physical Activity	B. K. Pogrmilovic, A. R. Varela, M. Pratt et al.	2020		*
Revista Brasileira de Atividade Física e Saúde	R. N.Silva, J. R. Oliveira, R. C. B. Carneiro et al.	2020	Brazil	Health Academy Program
Revista Brasileira de Atividade Física e Saúde	A. M. S. Ivo, V. C. Viana, M. I. F. Freitas	2020	Brazil	Health Academy Program
Revista Brasileira de Atividade Física e Saúde	R. C. F. Lima, B. L. S. Rodrigues, S. J. M. Farias et al.	2020	Brazil	Health Academy Program
Ciência & Saúde Coletiva	B. L. S. Rodrigues, R. N. Silva, R. G. Arruda et al.	2021	Brazil	Health Academy Program

International Journal of Environmental Research and Public Health	D. A. S. Silva e C. F. Silva	2022	Brazil	Health Academy, Health at School, New More Education, Second Time, Sports and Leisure in the City, Strengths in Sports, João do Pulo Project, Fight for Citizenship, Playing with Sports, Riverside Communities of the Amazon, DELAS, Sports and Citizenship, Turning the Game Around, Living Village, Selection of the Future, Initiation and improvement of sports, Pracinhas da Cultura
Health Promotion International,	M. A. Rubio, D. Mosquera, M. Blanco et al.	2022	Colombia	My Body
Saúde debate	P. M. C. Andrade, R. T. Silva, T. P. Pereira et al.	2022	Brazil	Health at School Program
Journal of Physical Activity and Health	J. M. Grueso, M. Pratt, E. Resendiz et al.	2024	Colombia, Ecuador	Colombia: 1. Ten-Year Public Health Plan (2022), 2. National Public Policy for the Development of Sport, Recreation, Physical Activity and the Use of Free Time towards a Territory of Peace (2018) / Ecuador: 3. Ten-Year Plan for Physical Culture of Ecuador—DEFIRE (2018), 4. Ten-Year Plan for Sport, Physical Education and Recreation—DEFIRE (2018) and 5. Student Participation Program (2016)
Revista Brasileira de Atividade Física e Saúde	F. G. Mallue, G. S. Leite, T. C. Dias et al.	2024	Brazil	Health at School Program

* information not included in the article; ** Colombian documents; *** Ecuadorian documents.
