# Supplementary material for: Studies Analyzing South American Public Policy Documents on Physical Activity: A Scoping Review
Source: Epidemiologia (Basel). 2026 Jun 29;7(4):89. doi: 10.3390/epidemiologia7040089 (PMC13397982; doi:10.3390/epidemiologia7040089)
Supplement: Supplementary file 1 [file epidemiologia-07-00089-s001.zip › Additional File S3_Scoping review_Ingrid.pdf]

Additional File S3 - Methodological aspects of studies that analyzed public policy documents on physical activity and/or sedentary behavior in South American countries (n=13).

Author	Year of publication	Document	Policy sector	Target	Strategy	Instrument	Analysis
A. G. Knuth, D. C. Malta, D. K. Cruz et al.	2010	National Health Promotion Policy	Health	General population	The coordinators received the questionnaires by mail and by email with a link to complete them electronically and had 60 days to respond to the questionnaire.	Questionnaire	Descriptive
R. N. Silva, F. R. B. Guarda, P. C. Hallal, P. J. L. Martelli	2017	Health Academy Program	Health	General population	Fourteen key informants were interviewed, four experts in the field of health program evaluation. They were asked to give their opinions on the components, perform the logical analysis and the consistency test of the logical model proposed for the program. And analysis of documents that regulate the Health Academy Program in Recife.	Semi-structured interview and questions to evaluate documents	Descriptive
R. Mora, M. Greene, M. Coradoc	2018	CicloRecreoVia Program	Health, Transport	General population	Program users responded to a questionnaire	Questionnaire	Descriptive
B. K. Pogrmilovic, A. R. Varela, M. Pratt et al.	2020	*	*	*	National contacts from 76 countries responded online	GoPA! Policy Inventory version 3.0 and Comprehensive Analysis of Policy on Physical Activity (CAPPA)	Descriptive and Inferential
R. N.Silva, J. R. Oliveira, R. C. B.	2020	Health Academy Program	Health	General population	Key Informant Interview	Interview	Descriptive

Carneiro et al.							
A. M. S. Ivo, V. C. Viana, M. I. F. Freitas	2020	Health Academy Program	Health	General population	Interviews with program users	Interview	Descriptive and Qualitative
R. C. F. Lima, B. L. S. Rodrigues, S. J. M. Farias et al.	2020	Health Academy Program	Health	General population	Secondary data from the SUS Information Technology Department (DATASUS) were analyzed.	The data was extracted with the help of DATASUS' own TABnet tool.	Inferential
B. L. S. Rodrigues, R. N. Silva, R. G. Arruda et al.	2021	Health Academy Program	Health	General population	Secondary data from the SUS Information Technology Department (DATASUS) were analyzed.	were collected from the website of the National Registry of Health Establishments (CNES), of the SUS Information Technology Department (DATASUS).	Descriptive and Inferential
D. A. S. Silva e C. F. Silva	2022	Health Academy, Health at School, New More Education, Second Time, Sports and Leisure in the City, Strengths in Sports, João do Pulo Project, Fight for Citizenship, Playing with	Health, Education, Tourism	Children and adolescents	Search all official websites of federal government agencies in Brazil to analyze all ongoing programs/actions at a national level.	Health Enhancing Physical Activity (HEPA) Policy Audit Tool (PAT)—version 2	Descriptive

		Sports, Riverside Communities of the Amazon, DELAS, Sports and Citizenship, Turning the Game Around, Living Village, Selection of the Future, Initiation and improvement of sports, Pracinhas da Cultura					
M. A. Rubio, D. Mosquera, M. Blanco et al.	2022	My Body	Health and leisure	Women	Interviews with program users	semi-structured interviews	Inferential
P. M. C. Andrade, R. T. Silva, T. P. Pereira et al.	2022	Health at School Program	Health, Education	Children and adolescents	Report analysis	secondary information, obtained through documentary sources from the Municipal Health and Education Departments and the Regional Education Management (GRE) of Vitória de Santo Antão-PE.	Descriptive

J. M. Grueso, M. Pratt, E. Resendiz et al.	2024	Colombia: 1. Ten-Year Public Health Plan (2022), 2. National Public Policy for the Development of Sport, Recreation, Physical Activity and the Use of Free Time towards a Territory of Peace (2018) / Ecuador: 3. Ten-Year Plan for Physical Culture of Ecuador—DEFIRE (2018), 4. Ten-Year Plan for Sport, Physical Education and Recreation—DEFIRE (2018) and 5. Student Participation Program (2016)	Health (1, 3), sport (2, 4), education (5)	General population (1-4); children, adolescents and young people (5)	Online contact with national and subnational government informants	Interaction between National and Local Government Levels in Development and Implementation of Physical Activity Policies Tool" (INTEGRATE-PA-Pol)	Descriptive
F. G. Mallue, G. S. Leite, T. C. Dias et al.	2024	Health at School Program	Health, Education	Children and adolescents	Program informants responded to a questionnaire	Questionnaire	Descriptive

* information not included in the article; ** only Ecuadorian documents.
