# Supplementary material for: Studies Analyzing South American Public Policy Documents on Physical Activity: A Scoping Review
Source: Epidemiologia (Basel). 2026 Jun 29;7(4):89. doi: 10.3390/epidemiologia7040089 (PMC13397982; doi:10.3390/epidemiologia7040089)
Supplement: Supplementary file 1 [file epidemiologia-07-00089-s001.zip › Additional File S4_Scoping review_Ingrid.pdf]

Additional File S4 - Objectives and main results of studies that analyzed public policy documents on physical activity and/or sedentary behavior in South American countries (n=13).

Author	Year of publication	Document	Objective	Main results
A. G. Knuth, D. C. Malta, D. K. Cruz et al.	2010	National Health Promotion Policy	Present the geographical distribution of all projects funded by the Ministry of Health from 2005 to 2008, as well as structure and process data on interventions funded in 2006 and 2007.	(1) Although the National Health Promotion Policy acts more incisively in the health and education departments, with intervention actions in physical activity, healthy eating and smoking prevention, there are notable urban actions in squares and sports courts. (2) The Central-West and North regions are those with the lowest number of funded projects; and most of the financed cities had less than 30,000 inhabitants.
R. N. Silva, F. R. B. Guarda, P. C. Hallal, P. J. L. Martelli	2017	Health Academy Program	Carry out an evaluability study of the Health Academy Program in the city of Recife, considering the description of the intervention, the elaboration of its logical and theoretical evaluation models, in addition to identifying evaluative questions"	The Health Academy Program presents a wide range of objectives, principles and guidelines, and some managers are unaware of these elements.
R. Mora, M. Greene, M. Coradoc	2018	CicloRecreoVia Program	Characterize the physical activity and mobility patterns of participants in the CicloRecreoVia program in Santiago	People use the CicloRecreoVia program for leisure, recreation and health purposes. Regardless of the purpose, they can meet the WHO recommendations for weekly physical activity.
B. K. Pogrmilovic, A. R. Varela, M. Pratt et al.	2020	*	Audit and critically evaluate the availability, comprehensiveness, implementation and effectiveness of current physical activity and sedentary	The Americas are among the regions with the most physical activity policies (+90%) and around 60% of policies to address sedentary behavior

			behavior policies at national and global levels.	
R. N.Silva, J. R. Oliveira, R. C. B. Carneiro et al.	2020	Health Academy Program	To assess the degree of implementation of the Health Academy Program in the city of Bezerros, Pernambuco	In the city of Bezerros, the level of implementation of the Health Academy Program is considered intermediate (55.0%), with the structure dimension obtaining a higher score (64.7%) than the work process dimension (48.6%).
A. M. S. Ivo, V. C. Viana, M. I. F. Freitas	2020	Health Academy Program	Understand the meaning of physical activity and the Health Academy Program, according to users, to discuss the consequences of the Previne Brasil Program in the structuring and operation of the Program.	The Health Academy Program has favored users' understanding of the health-disease process in a health promotion model that seeks to empower individuals, with comprehensive care and autonomy for healthy lifestyle choices.
R. C. F. Lima, B. L. S. Rodrigues, S. J. M. Farias et al.	2020	Health Academy Program	To assess the impact of the Health Academy Program on hospitalization costs for Cerebrovascular Diseases in the state of Pernambuco	Cities that do not have the Health Academy Program implemented have higher expenses with hospital admissions due to Cerebrovascular Diseases, compared to cities that have the program's actions.
B. L. S. Rodrigues, R. N. Silva, R. G. Arruda et al.	2021	Health Academy Program	To assess the impact of the Academia da Saúde Program on mortality due to Systemic Arterial Hypertension in the state of Pernambuco, Brazil	The presence of the Academia da Saúde Program resulted in a 12.8% reduction in the mortality rate due to high blood pressure, mainly among people of mixed race and those over 80 years of age.
D. A. S. Silva e C. F. Silva	2022	Health Academy, Health at School, New More Education, Second Time, Sports and Leisure in the City, Strengths in	Investigate the characteristics of national physical activity policies in Brazil through actions and programs to promote physical activity for children and adolescents	No actions/strategies were found that focused solely on reducing physical inactivity and sedentary behavior among adolescents.

		Sports, João do Pulo Project, Fight for Citizenship, Playing with Sports, Riverside Communities of the Amazon, DELAS, Sports and Citizenship, Turning the Game Around, Living Village, Selection of the Future, Initiation and improvement of sports, Pracinhas da Cultura		
M. A. Rubio, D. Mosquera, M. Blanco et al.	2022	My Body	Evaluate the networking process to co-create the My Body physical activity program	The co-creation of My Body is possible through intersectoral actions, successful dissemination of evidence, and increased relationships and communications between stakeholders.
P. M. C. Andrade, R. T. Silva, T. P. Pereira et al.	2022	Health at School Program	Describe the scope of the Health in Schools Program and the main themes addressed in the practices carried out in Vitória de Santo Antão-PE	In Vitória de Santo Antão, the School Health Program does not have a comprehensive scope and, when implemented in rural areas, the actions of anthropometric assessment, vaccination status, identification of possible signs directed at neglected diseases (leprosy and worms) stood out, and in urban areas, anthropometric assessment, oral health and early detection of Systemic Arterial Hypertension (SAH).
J. M. Grueso, M. Pratt, E.	2024	Colombia: 1. Ten-Year Public	Characterize the development and implementation process of	Colombia's policies integrate actions at all levels, one of which has the promotion of physical activity as its

Resendiz et al.		Health Plan (2022), 2. National Public Policy for the Development of Sport, Recreation, Physical Activity and the Use of Free Time towards a Territory of Peace (2018) / Ecuador: 3. Ten-Year Plan for Physical Culture of Ecuador—DEFIRE (2018), 4. Ten-Year Plan for Sport, Physical Education and Recreation—DEFIRE (2018) and 5. Student Participation Program (2016)	national and subnational physical activity policies, to better understand coordination between levels	central objective. And Ecuador's policies, the national ones do not communicate with the local ones and understand the promotion of physical activity as important, they are not central.
F. G. Mallue, G. S. Leite, T. C. Dias et al.	2024	Health at School Program	To describe the existence of the Health at School Program in 2022 in municipal schools in Pelotas, Rio Grande do Sul, the actions developed and the perspectives of education	Actions related to the School Health Program predominantly involve actions to promote and assess oral health, promote a culture of peace, citizenship and human rights, and combat the Aedes aegypti mosquito.

			professionals on the implementation of the Program.	

* information not included in the article; ** Colombian documents; *** Ecuadorian documents.
